# Genome-wide association study of serum magnesium in type 2 diabetes

**DOI:** 10.1186/s12263-024-00738-5

**Published:** 2024-01-26

**Authors:** Lynette J. Oost, Roderick C. Slieker, Marieke T. Blom, Leen M. ’t Hart, Joost G. J. Hoenderop, Joline W. J. Beulens, Jeroen H. F. de Baaij

**Affiliations:** 1 Department of Medical BioSciences, Radboud University Medical Center, Nijmegen, the Netherlands; 2 Department of Epidemiology and Data Science, Amsterdam UMC, Vrije Universiteit, Amsterdam, Amsterdam, the Netherlands; 3 Department of Cell and Chemical Biology, Leiden University Medical Center, Leiden, the Netherlands; 4 Amsterdam Public Health, Health Behaviors and Chronic Diseases, Amsterdam, the Netherlands; 5 Amsterdam Cardiovascular Sciences, Diabetes & Metabolism, Amsterdam, the Netherlands; 6 Department of General Practice, Amsterdam UMC, Vrije Universiteit Amsterdam, Amsterdam, the Netherlands; 7 Department of Biomedical Data Sciences, Section Molecular Epidemiology, Leiden University Medical Center, Leiden, the Netherlands

**Keywords:** GWAS, Type 2 diabetes mellitus, Mg^2+^, Hypomagnesemia, Single nucleotide polymorphism

## Abstract

People with type 2 diabetes have a tenfold higher prevalence of hypomagnesemia, which is suggested to be caused by low dietary magnesium intake, medication use, and genetics. This study aims to identify the genetic loci that influence serum magnesium concentration in 3466 people with type 2 diabetes. The GWAS models were adjusted for age, sex, eGFR, and HbA1c. Associated traits were identified using publicly available data from GTEx consortium, a human kidney eQTL atlas, and the Open GWAS database. The GWAS identified a genome-wide significant locus in *TAF3* (*p* = 2.9 × 10^−9^) in people with type 2 diabetes. In skeletal muscle, loci located in *TAF3* demonstrate an eQTL link to *ATP5F1C*, a gene that is involved in the formation of Mg^2+^-ATP. Serum Mg^2+^ levels were associated with *MUC1/TRIM46* (*p* = 2.9 × 10^−7^)*, SHROOM3* (*p* = 4.0 × 10^−7^), and *SLC22A7* (*p* = 1.0 × 10^−6^) at nominal significance, which is in combination with the eQTL data suggesting that they are possible candidates for renal failure. Several genetic loci were in agreement with previous genomic studies which identified *MUC1/TRIM46* (*P*_meta_ = 6.9 × 10^−29^, *P*_Q_ = 0.81) and *SHROOM3* (*P*_meta_ = 2.9 × 10^−27^, *P*_Q_ = 0.04) to be associated with serum Mg^2+^ in the general population. In conclusion, serum magnesium concentrations are associated with genetic variability around the regions of *TAF3, MUC1/TRIM46, SHROOM3*, and *SLC22A7* in type 2 diabetes.

**Supplementary Information:**

The online version contains supplementary material available at 10.1186/s12263-024-00738-5.

## Introduction

Magnesium (Mg^2+^) is an essential ion that is involved in more than 600 enzymatic reactions, including DNA synthesis and energy metabolism [1]. Consequently, Mg^2+^ levels are tightly regulated by the interplay of kidney, intestine, bone, and soft tissues [1]. The serum concentration of Mg^2+^ is partly explained by genetic factors with a heritability estimate ranging from 15 to 39% [2]. GWAS performed in the healthy population identified genetic variants in *MUC1, TRPM6*, and *SHROOM3* loci to be associated with serum Mg^2+^ concentration [3–5].

The prevalence of hypomagnesemia is between 10 and 45%, which is 10 times higher in people with type 2 diabetes mellitus compared to the healthy population [6–8]. Serum Mg^2+^ is inversely associated with HbA_1c_ in people with type 2 diabetes. The inverse association of serum Mg^2+^ with insulin levels is found in people with and without diabetes [7, 9, 10]. Since hypomagnesemia is associated with poor glycemic control and insulin resistance, it leads to a higher incidence of type 2 diabetes and worsens existing type 2 diabetes [11, 12]. This mechanism may explain why genetic variants in Mg^2+^-related ion channels have been linked to diabetes risk [13]. For example, genetic variants in *TRPM6* are associated with the development of gestational diabetes and type 2 diabetes [14, 15] and SNPs in *CNNM2, SLC41A2*, and *TRPM6* were associated with the risk of diabetes through serum Mg^2+^ levels [11].

Several causes for hypomagnesemia have been described in people with type 2 diabetes including low dietary Mg^2+^ intake, dyslipidemia, medication use, and genetics. [3, 7, 11, 14] Given the multifactorial pathophysiological causes of type 2 diabetes, including insulin resistance, low-grade chronic inflammation, and dyslipidemia, additional genetic factors may contribute to hypomagnesemia in type 2 diabetes [16]. People with type 2 diabetes often have hypermagnesiuria, which is a fractional excretion of Mg^2+^ (FEMg) above 4%, which is suggested to be caused by reduced Mg^2+^-channel TRPM6 activity [14, 15, 17, 18]. Hypermagnesiuria in the presence of hypomagnesemia suggests that urinary Mg^2+^ wasting resulting from reduced reabsorption in the kidneys is a major cause of hypomagnesemia in people with type 2 diabetes [6, 19]. However, it is unknown whether genetic variants in people with type 2 diabetes contribute to urinary Mg^2+^ wasting. Gene variants that determine serum Mg^2+^ levels in people with type 2 diabetes have never been systematically studied. The genetic variation of serum Mg^2+^ in type 2 diabetes compared to the healthy population can differ and help prevent the development of associated adverse clinical outcomes, such as heart failure and microvascular disease [20]. Therefore, we aimed to determine the genetic variations in serum Mg^2+^ specifically for people with type 2 diabetes.

## Materials and methods

### Study population

The Hoorn DCS is a prospective observational study of individuals ﻿with diabetes defined as (1) at least one symptom of excessive thirst, polyuria, weight loss, hunger, or pruritus combined with fasting plasma glucose ≥ 7.0 mmol/L or random plasma glucose ≥ 11.1 mmol/L, or (2) two elevated plasma glucose concentrations on two different occasions in the absence of symptoms. The baseline biobanking was done in the years 2008–2009 and 2012–2014. Further details of this cohort are published by van der Heijden et al. [21].

Ethical approval was obtained from the VU University Medical Center Ethical Review Committee (09/07/2009, ref: NL27783.029.09), Amsterdam. All participants gave written informed consent before participation in the Hoorn DCS biobank.

### Serum magnesium measurement

Serum samples of 4445 people were measured for Mg^2+^, of which 45 were stored/collected in EDTA tubes or had insufficient volumes and were therefore excluded. Furthermore, 52 people were excluded due to a different type of diabetes. From the 4348 participants that were left, 882 participants were excluded because GWAS data was not available. Mg^2+^ was measured in 2019 at the Laboratory Medicine Department (Radboud University Medical Center) using a calibrated standardized colorimetric assay with a coefficient of variation of 1.98% (Cobas C8000; Roche Diagnostics, Risch-Rotkreuz, Switzerland). Additional information is published elsewhere by Oost et al. [20]. Missing data was for all variables < 5%.

### Statistical methods for genome-wide association study

Genotypes were determined with the Illumina Human Core Exome array. Principal component analysis was performed with plink. Genotypes were imputed using the Michigan Imputation Server based on the Haplotype Reference Consortium (HRC) panel.

GWAS on serum Mg^2+^ concentration was performed using rvtest (version 20170613) on an additive model on 39127679 SNPs. Principal components (PCs) were included to adjust for the potential effects of population stratification. SNPs with an allele frequency below 0.05 or with low imputation confidence (info score < 0.3) were filtered out. After filtering, 5407047 remained in the final set. A *p* value below 5 × 10^−8^ was considered genome-wide significant. Three models were explored to evaluate the association between genetics and serum Mg^2+^: base model adjusted for age, sex, and the first three PCs (1–3), model 1 additionally adjusted for eGFR, and model 2 additionally adjusted for eGFR and HbA1c. Models 1 and 2 were adjusted for eGFR combined with HbA1c because eGFR is a measure of kidney function and HbA1c is a measure of the severity of type 2 diabetes. Additionally, serum Mg^2+^. Hence, the variability of glycemic genetic factors may cause fluctuations in serum Mg^2+^.

After identification of the lead SNPs, expression quantitative trait loci (eQTL) and associated traits were assessed using publicly available data from the genotype-tissue expression (GTEx) consortium (v8), the human kidney eQTL atlas from the Susztak lab (meQTL_S443-eQTM_S414) and the Open GWAS database (v7.5.12) [22–24]. For comparison to previous GWAS, we downloaded the identified top hits identified by Meyer et al. [3]. Loci were meta-analyzed using GWAMA (v2.1) based on the current data and Meyer et al. Meta-analyzed results and Cochran’s *Q* test were obtained for overlapping loci. For *ATP2B1* we used a proxy SNP because the original SNP was not available in our own dataset.

A power calculation was performed using the R package *genpwr* with the following settings: *N* = 3466, sd_y = 0.0789, MAF = 0.05, 0.10, effect size = 0.005, 0.01, 0.015, 0.02, alpha = 0.05, additive model.

## Results

In 3466 participants, the mean serum Mg^2+^ was 0.80 ± 0.08 mmol/L and 330 individuals (9.5%) showed hypomagnesemia (serum Mg^2+^ < 0.7 mmol/L). All of these people were of European descent. Other participant characteristics are reported in Table 1.

**Table 1:** Baseline characteristics of individuals with GWAS data available in the Hoorn, DCS study (*n* = 3466)

Demographics	
Men (%)	1973 (57)
Age (years)	66 ± 10
Duration of diabetes (years)	5.6 (3.0–10.1)
Smoking, *n* (%)	
Current	609 (18)
Former	1776 (51)
Never	1058 (31)
Missing	23 (1)
Metabolic variables	
BMI (kg/m^2^)	29.6 (26.7–33.2)
SBP (mmHg)	141 ± 20
DBP (mmHg)	78 ± 9
Total cholesterol (mmol/L)	4.6 ± 1.0
Triglycerides (mmol/L)	1.6 (1.1–2.1)
HbA1c (%)	6.5 (6.1–7.1)
HbA1c (mmol/mol)	47.5 (43.0–54.0)
Fasting glucose (mmol/L)	7.7 (6.9–8.9)
LDL (mmol/L)	2.5 ± 0.9
HDL (mmol/L)	1.2 (1.0–1.4)
Albumin creatine ratio (mg/mmol)	0.5 (0.0–1.3)
Serum creatinine (μmol/L)	78 (67–91)
eGFR (mL/min/1.73m^2^)	79.5 ± 18.3
Serum Mg^2+^ (mmol/L)	0.80 ± 0.08
Medication use, *n* (%)	
Insulin	743 (21)
Glucose-lowering medication	2628 (76)
Diuretics	1040 (30)

Characteristics are presented as *n* (%), or mean ± SD, or median (interquartile range). Mg^2+^ in mmol/L. *BMI* = body mass index, *DBP* = diastolic blood pressure, *DCS* = Diabetes Care System, *eGFR* = estimated glomerular filtration rate, *HbA1c* = hemoglobin A1c, *HDL* = high density-lipoprotein cholesterol, *LDL* = low density, *Mg*^*2+ *^*=* magnesium ion, *SBP* = systolic blood pressure

### Magnesium GWAS

**Fig. 1:**
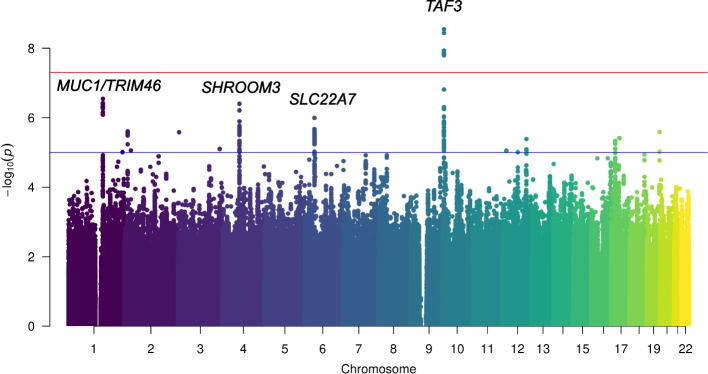
Genome-wide –log10(p-value) plots from association analyses with serum Mg^2+^ concentration in 3466 people with type 2 diabetes in the Hoorn, DCS study. Adjusted for age, sex, and PC1-3. One locus *(TAF3,* rs7894336*)* reached genome-wide significance (*P* < 5 × 10^−8^), indicated by the red horizontal line. Three loci (*TRIM46*, rs11264341; *SHROOM3*, rs10019833, and *SLC22A7*, rs2270860) reached nominal significance (*P* < 10−6), indicated by the blue horizontal line. DCS=Diabetes Care System, Mg^2+^ = magnesium, *PC* = principal component, *T2D* = type 2 diabetes

**Fig. 2:**
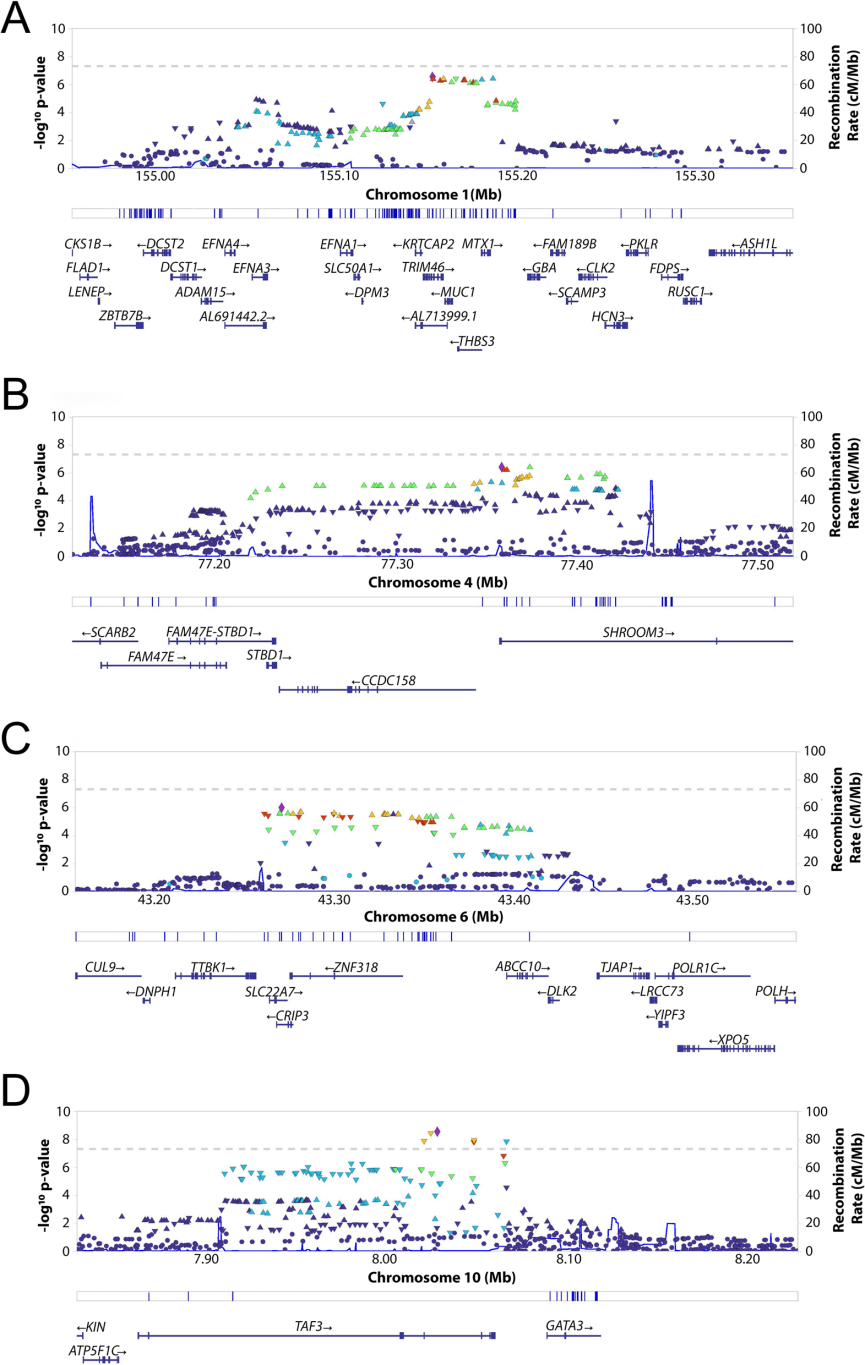
Regional locus association plot showing −log10 (P values) for the serum Mg^2+^-associated regions near (**A**) *TRIM46* and *MUC1* gene on chr1, (**B**) *SHROOM3* and *CCDC158* gene on chr4, (**C**) *SLC22A7* and *ZNF318* gene on chr6, and (**D**) *TAF3* gene on chr10. Top panel shows the *P* value (−log10) for each SNP. The colors of the points indicate the linkage disequilibrium with the top signal indicated by the diamond. The blue line represents the recombination rate. The middle panel shows the location of the SNP indicated by a blue line and the lower panel shows the genes in the region. *Chr* = chromosome, *Mg*^*2+*^ = magnesium

Figure 1 shows the Manhattan plot for associations between SNPs and serum Mg^2+^ concentration after adjustment for age, sex, and PC 1–3. The *Q*-*Q* plots of the observed versus expected *p* value distributions for associations between the SNPs and Mg^2+^ are provided in Supplementary Figure S1. The lowest *p* value within the region (lead SNP) that reached genome-wide significance (*p* < 5 × 10^−8^) was located in the *TAF3* gene (rs7894336, chromosome (chr)10, *p* = 2.86 × 10^−9^). Other lead SNPs with nominal significance (equal to or below *p* < 10^−6^) were located near *MUC1/TRIM46* (rs11264341, chr1, *p* = 2.9 × 10^−7^), *SHROOM3* (rs10019833, chr4, *p* = 4.0 × 10^−7^), and *SLC22A7* (rs2270860, chr6, *p* = 1.0 × 10^−6^). Regional association plots (LocusZoom) for chr1, 4, 6, and 10 are shown in Fig. 2A–D.

Adjusting the serum Mg^2+^-SNP associations for eGFR did not change the association with serum Mg^2+^ levels (Supplementary Figure S2A and Supplementary Table S1). Adjusting the Mg^2+^-SNP associations for eGFR and HbA1c, demonstrated a stronger significance only at loci SLC22A7 (rs2270860, chr6) from *p* = 1.3 × 10^−6^ to *p* = 5.6 × 10^−8^ (Supplementary Figure S2B and Supplementary Table S1). Loci identified in a previous GWAS did show similar effect sizes. Although they were not significant in DCS, six out of the nine loci were significant when meta-analyzed (Supplementary Table S2). Heterogeneity between cohorts was observed for two loci (*SHROOM3* and *TRPM6, Q_P*-value *= 0.035~0.043*), but this was based on differences in the magnitude of effect and not the direction of effect.

### eQTL studies using GTEx data

The lead genetic variant rs7894336 (chr10, *TAF3*) had no expression quantitative trait loci (eQTL) associations. SNPs in or near the *TAF3* locus were associated with *ATP5F1C* expression in skeletal muscle (rs10795574, chr10, *p* = 9.4 × 10^−7^) (Supplementary Table S3). On chromosome 10, *ATP5F1C* is located near *TAF3* (Fig. 2D). *ATP5F1C* encodes the gamma subunit of the catalytic core F1 of the mitochondrial ATP synthase and creates a proton gradient for ATP synthesis [25]. Mg^2+^ is known to bind to the catalytic F1 unit to produce the high-energy terminal bond of ATP (Mg^2+^-ATP), which is essential in glycolysis [1, 26].

rs11264341 (chr1 near *MUC1/TRIM46*) had significant associations with *GBAP1* expression in multiple tissues, including artery tibial (*p* = 2.1 × 10^−30^), artery aorta (*p* = 1.76 × 10^−25^), heart left ventricle (*p* = 7.0 × 10^−25^) and lung (*p* = 2.7 × 10^−20^). Other associations of rs11264341 with *MUC1* and *THBS3* were most significant in esophagus mucosa tissue (*p* = 1.8 × 10^−21^) and whole blood (*p* = 7.6 × 10^−20^), respectively. rs10019833 (chr4, *SHROOM3*) had significant associations with *FAM47E* in thyroid tissue (*p* = 2.1 × 10^−27^), *STBD1* in nerve tibial tissue (*p* = 5.8 × 10^−21^) and *CCDC158* in artery tibial tissue (*p* = 3.1 × 10^−18^). rs2270860 (chr6, *SLC22A7*) had significant associations with *SLC22A7* in testis (*p* = 2.5 × 10^−17^), *ZNF318* in adrenal gland tissue (*p* = 1.6 × 10^−9^) and *CRIP3* in left ventricle heart tissue (*p* = 3.0 × 10^−7^). An overview of the information on the lead SNPs within each chromosomal region is presented in Table 2 and eQTL links from the genotype-tissue expression (GTEx) consortium in Table 3.

**Table 2:** Base model of associations between serum Mg^2+^ concentrations and the lead regional genome-wide significant SNPs.

SNP	chr	Location	Allele frequency^a^	Function	Coded^b^	Closest gene	% Variance explained	Beta (mmol/L)	SE	*P*
rs7894336	10	8026609	0.53	*Intronic*	C>T	*TAF3*	0.54	− 0.011	0.002	2.9E−9
rs11264341	1	155151493	0.47	*Intronic*	C>T	*TRIM46*	0.44	0.010	0.002	2.9E−7
rs10019833	4	77357592	0.37	*Intronic*	T>C	*SHROOM3*	0.41	0.010	0.002	4.0E−7
rs2270860	6	43270151	0.31	*Synonymous*	C>T	*SLC22A7*	0.28	− 0.010	0.002	1.0E−6

Base model is adjusted for age, sex, and PC 1-3

*chr* = chromosome, *Mg*^*2+*^ = magnesium ion, *PC* = principal component, *SE* = standard error, *SNP* = single nucleotide polymorphism

^a^Allele frequency is for European populations, according to the gnomAD database v3.1.2. [27]

^b^Coded alleles are inversely associated with serum Mg^2+^

**Table 3:** Loci associated with genetic variability according to GTEx consortium in all human tissues available and human kidney eQTL meta-analysis

SNP	chr	Location	Coded	Linked gene^1^	NES^1^	*P*^1^	Linked gene^2^	Beta^2^	STD^2^	*P*^2^
rs11264341	1	155151493	C>T	*GBAP1*	− 0.404	2.0E-30	*MUC1*	0.176	0.024	1.7E−13
rs10019833	4	77357592	T>C	*FAM47E*	− 0.324	2.1E-27	*STBD1*	0.207	0.041	5.5E−7
rs2270860	6	43270151	C>T	*SLC22A7*	− 0.313	2.5E-17	*CRIP3*	0.221	0.050	1.1E−5

*GTEx* = genotype-tissue expression, *Mg*^*2+*^ = magnesium ion, *NES* = normalized affect size, *STD* = standard deviation

^1^eQTL looked up in the GTEx consortium [28]

^2^Data was retrieved from https://susztaklab.com/Kidney_eQTL/pub.php [24]

^3^Beta is reported with respect to the alternate allele

### eQTL and DNA methylation studies using the human kidney eQTL atlas from the Susztaklab and the Open GWAS database

Since serum Mg^2+^ concentration is primarily controlled by the kidney, significant genetic variability was mapped to human traits in kidney tissue using the human kidney eQTL atlas from the Susztak lab [24]. In kidney tissue, no eQTLs associations of rs7894336 (chr10, *TAF3*) were shown. The three lead SNPs with nominal significance (all *p* ≤ 10^−6^) did show significant cis associations in human kidney tissue. rs11264341 (chr1 near *MUC1/TRIM46*) had a significant association with *MUC1* (rs11264341*,* chr1, *p* = 1.7 × 10^−13^), rs10019833 (chr4, *SHROOM3*) had a significant association with *STBD1* (chr4, *p* = 5.5 × 10^−7^) and rs2270860 (chr6, *SLC22A7*) had a significant association with *CRIP3* (rs2270860, chr6, *p* = 1.1 × 10^−5^) in human kidney tissue. The human kidney methylation quantitative trait loci atlas of the Susztak-lab showed associations of nominal significant lead SNPs with *EFNA3, MUC1, STBD1, SHROOM3, POLH*, and *SLC22A7* (Supplementary Table S4). The Open GWAS database showed the lead genetic variant rs7894336 (chr10, *TAF3*) association with phosphate. The nominal significant lead SNP rs11264341 (chr near *MUC1/TRIM46*) was associated with urea, while rs10019833 (chr4, *SHROOM3*) and rs2270860 (chr6, *SLC22A7*) were associated with cystatin C (Supplementary Table S5) [23].

## Discussion

We report the first GWAS of serum Mg^2+^ in 3466 individuals with type 2 diabetes. We did have 80% power to identify variants with a MAF of 5% and an effect of 0.012 and 80% power to identify variants with a MAF of 10% and an effect of 0.009. The association of the *TAF3* locus with the serum Mg^2+^ concentration reached genome-wide significance in the base model and the eGFR- and eGFR/HbA1c-adjusted models. *MUC1*/*TRIM46, SHROOM3,* and *SLC22A7* loci were associated with serum Mg^2+^ concentration at nominal significance*.* We linked serum Mg^2+^-associated SNPs with *MUC1, STBD1,* and *CRIP3* in kidney tissue. The lead SNP in the *TAF3* region was associated with *ATP5F1C* in skeletal muscle.

TAF3 is a general transcription factor involved in histone modification and gene expression of the tumor suppressor p53 [29]. Genetic variability in *TAF3* was linked to *ATP5F1C* expression in muscle. ATP5F1C encodes the gamma-subunit of the mitochondrial ATP Synthase, which is essential in the formation of Mg^2+^-ATP. In skeletal muscle, we also identified an eQTL for *RPL7L1,* which is predicted to enable RNA binding activity and structural constituent of the ribosome. This suggests that genetic variability in serum Mg^2+^ may be related to genes involved in transcription, translation, and ATP synthesis. Interestingly, genetic variability in *GATA3*, a region located near *TAF3*, was associated with serum calcium (Ca^2+^) levels in the general population (5). However, we did not find a genetic link to *GATA3* based on eQTL data.

In this study, we also found nominal significant genetic variability in *MUC1/TRIM46*, of which the lead SNP (rs11264341, chr1) was negatively associated with serum uric acid levels in a previously published GWAS [30]. Interestingly, increased Mg^2+^ intake is associated with a decreased risk of hyperuricemia [31]. Furthermore, the accumulation of uric acid can lead to kidney stone formation, oxidative stress, insulin resistance, and increased type 2 diabetes incidences [32]. Although the same lead SNP (rs11264341) in *MUC1*/*TRIM46* did not provide evidence of a causal relationship between serum uric acid and incident diabetes, in people without a history of diabetes [33]. In our study, there was an eQTL link of the lead SNP with *GBAP1* in multiple tissues and with *MUC1* in kidney tissue. The association of serum Mg^2+^ with the *MUC1/TRIM46* locus is also found in all previously published GWAS on serum Mg^2+^ concentration performed in the general population [3–5]. MUC1 is a membrane-bound glycosylated phosphoprotein that is attached to the apical surface of epithelial cells in the intestinal tract and plays a critical in mucosal defense by preventing the binding of pathogens [34].

Genetic variability in *SHROOM3* (chr4) is associated with serum Mg^2+^ levels in GWAS based on the general population [3, 5]. Furthermore, intronic *SHROOM3* genetic variants in multiple GWAS have been associated with chronic kidney disease (CKD) and kidney function markers: eGFR and creatinine levels [35, 36]. In kidney tissue, the lead SNP is linked to *STBD1* expression. STBD1 encodes starch binding domain 1, which plays an important role in the transport of glycogen to lysosomes [37]. *STBD1* is identified in a GWAS meta-analysis that prioritized target genes for kidney diseases [24]. Animal studies have shown that disruption of *SHROOM3* causes podocyte effacement and impairment of the glomerular filtration barrier [38], demonstrating that this genetic loci is important for kidney health. On chromosome 6, we identified genetic variability in *SLC22A7* in the association with serum Mg^2+^. Still, a lot is unknown about the function of SLC22A7, but previous studies report that it is involved in the transport of cyclic nucleotide cGMP, renal excretion, and possibly creatine reabsorption in renal proximal tubular cells [39, 40]. The cGMP signaling pathway does induce Mg^2+^ release [41], and creatinine clearance is an important measure of renal function.

According to the Open GWAS database, all genetic variants identified in this study were associated with markers of kidney function; like phosphate, urea, and cystatin C [23]. The kidney plays a role in the volume and mineral balance and therefore is an essential regulator of the serum Mg^2+^ concentration [42]. Nevertheless, we exclude kidney function as an explanation for the association between the SNPs and serum Mg^2+^, since adjusting the serum Mg^2+^-SNP associations for eGFR did not change the association.

Previous studies have identified serum Mg^2+^-associated genetic variants (rs1114413 or rs113607577 or rs3824347, chr9) near the *TRPM6 gene*, but in the current study, we did not replicate this finding [3, 4, 43]. A possible explanation is that our cohort consists mainly of insulin-resistant individuals. In healthy people, insulin would regulate *TRPM6* channel activity [15], but this may be impaired in diabetes. Based on the eQTL associations and Open GWAS database, all genetic variants are linked to disturbance in Mg^2+^ absorption and markers of renal failure. Interestingly, our eGFR-adjusted analyses did not alter the associations of the identified genetic variations with serum Mg^2+^. However, a large majority of the cohort (approximately 25%) already had a low eGFR (< 60 mL/min/1.73 m^2^) [20], suggesting that adjustment for eGFR does not have pronounced effects on the association of these genetic variants with serum Mg^2+^. This would suggest that the genetic variants identified are associated with markers of renal failure, which may reduce Mg^2+^ absorption in the kidneys.

This study has several strengths and limitations. One limitation of this study is that all eQTL and mQTL associations are obtained from databases that used tissue samples from the general population. There is a possibility that these associations are not present in tissue samples from people with type 2 diabetes. A second limitation is that 3,466 participants are a relatively small population for a GWAS. Despite the relatively small population size, many associations were below nominal significance *p* < 10^−6^, suggesting that the current study has sufficient power to find stronger signals. The population size was unfortunately too small to perform a causal analysis (i.e., Mendelian randomization analysis) to assess the relationship between serum Mg^2+^ and health complications of type 2 diabetes. Larger type 2 diabetes GWAS studies that measured serum Mg^2+^ are warranted to confirm our results and to perform causal analysis, however, to the best of our knowledge these are not available yet.

In this study, we have discovered novel loci that are associated with serum Mg^2+^ in people with type 2 diabetes. Our results suggest that genetic variation in or near *TAF3*, *MUC1/TRIM46, SHROOM3*, and *SLC22A7* are associated with the regulation of serum Mg^2+^ concentrations which may be partially explained by renal function, in type 2 diabetes. Genetic variation may explain why certain individuals with type 2 diabetes are at risk of developing hypomagnesemia.

### Supplementary Information

**Additional file 1: Supplementary Figure S1.** Q-Q plots showing the distribution of observed versus expected −log10(pvalues) for Mg2+ in (A) base model - corrected for age, sex and PC1-3 - genomic inflation factor 1.003277, (B) model 1- corrected for age, sex, eGFR, PC1-3 - genomic inflation factor 1.003411, and (C) model 2 - corrected for age, sex, eGFR, HbA1c, PC1-3 - genomic inflation factor 1.005874, in the Hoorn DCS cohort. **Supplementary Figure S2.** Genome-wide –log10(p-value) plots from association analyses with serum Mg2+ concentration in 3,466 people with type 2 diabetes in the Hoorn, DCS study. (A) Adjusted for age, sex, PC 1-3 and eGFR and (B) Adjusted for age, sex, PC 1-3, eGFR and HbA1c. Genome-wide significance (*P*<5×10^−8^) is indicated by the red horizontal line. The blue line presents significance *p<*10^−6^. DCS=Diabtetes Care Ssytem, eGFR=estimated glomerular filtration rate, HbA_tc_=hemoglobin A_tc_, PC=principal component. **Supplementary Table S1.** Adjusted models of associations between serum Mg2+ concentrations and the lead regional genome-wide significant SNPs. **Supplementary Table S2.** Loci that display similar effect sizes and an identical direction of the effect on serum Mg2+ levels in people with type 2 diabetes (DCS cohort) and in a previous study focused on the general population. **Supplementary Table S3.** Loci associated with genetic variability according to GTEx consortium in skeletal muscle tissue. **Supplementary Table S4.** Loci associated with genetic variability according to human kidney meQTL and eQTM association analyses. **Supplementary Table S5.** Traits associated with genetic variability according to the Open GWAS database

**Additional file 2.**

**Additional file 3.**

**Additional file 4.**

## Data Availability

The data supporting the conclusions of this research are reported within the article and its supplementary files, including the summary statistics files. Individual data cannot be made public due to privacy issues.
