# Supplementary material for: Genome-wide association study of serum magnesium in type 2 diabetes
Source: Genes Nutr. 2024 Jan 26;19:2. doi: 10.1186/s12263-024-00738-5 (PMC10811844; doi:10.1186/s12263-024-00738-5)
Supplement: Supplementary file 1 — Additional file 1: Supplementary Figure S1. Q-Q plots showing the distribution of observed versus expected −log10(pvalues) for Mg2+ in (A) base model - corrected for age, sex and PC1-3 - genomic inflation factor 1.003277, (B) model 1- corrected for age, sex, eGFR, PC1-3 - genomic inflation factor 1.003411, and (C) model 2 - corrected for age, sex, eGFR, HbA1c, PC1-3 - genomic inflation factor 1.005874, in the Hoorn DCS cohort. Supplementary Figure S2. Genome-wide –log10(p-value) plots from association analyses with serum Mg2+ concentration in 3,466 people with type 2 diabetes in the Hoorn, DCS study. (A) Adjusted for age, sex, PC 1-3 and eGFR and (B) Adjusted for age, sex, PC 1-3, eGFR and HbA1c. Genome-wide significance (P<5×10^−8^) is indicated by the red horizontal line. The blue line presents significance p<10−6. DCS=Diabtetes Care Ssytem, eGFR=estimated glomerular filtration rate, HbAtc=hemoglobin Atc, PC=principal component. Supplementary Table S1. Adjusted models of associations between serum Mg2+ concentrations and the lead regional genome-wide significant SNPs. Supplementary Table S2. Loci that display similar effect sizes and an identical direction of the effect on serum Mg2+ levels in people with type 2 diabetes (DCS cohort) and in a previous study focused on the general population. Supplementary Table S3. Loci associated with genetic variability according to GTEx consortium in skeletal muscle tissue. Supplementary Table S4. Loci associated with genetic variability according to human kidney meQTL and eQTM association analyses. Supplementary Table S5. Traits associated with genetic variability according to the Open GWAS database [file 12263_2024_738_MOESM1_ESM.pdf]

SUPPLEMENTARY MATERIAL

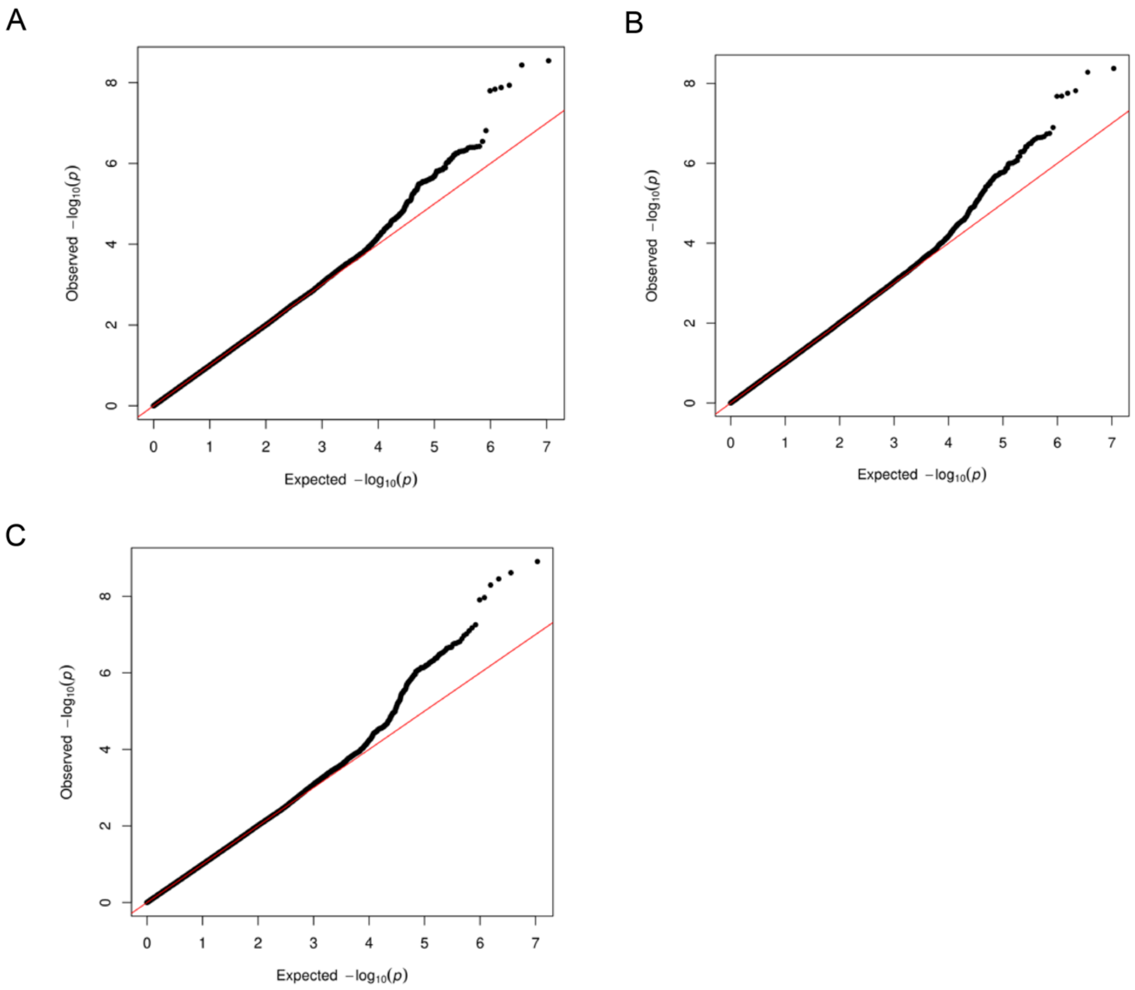

Supplementary Figure 1. Q-Q plots showing the distribution of observed versus expected −log10(p-values) for Mg^{2+} in (A) base model – corrected for age, sex and PC1-3 - genomic inflation factor 1.003277, (B) model 1- corrected for age, sex, eGFR, PC1-3 - genomic inflation factor 1.003411, and (C) model 2 - corrected for age, sex, eGFR, HbA_{1c}, PC1-3 - genomic inflation factor 1.005874, in the Hoorn DCS cohort.

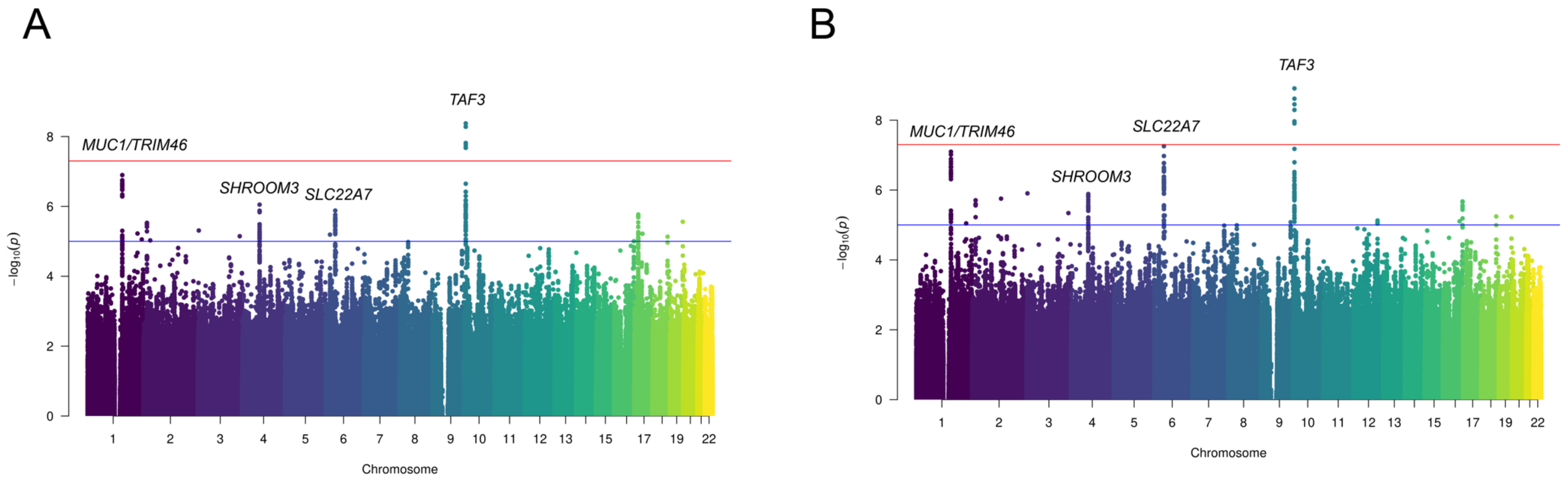

Supplementary Figure 2. Genome-wide –log10(p-value) plots from association analyses with serum Mg^{2+} concentration in 3,466 people with type 2 diabetes in the Hoorn, DCS study. (A) Adjusted for age, sex, PC 1-3 and eGFR and (B) Adjusted for age, sex, PC 1-3, eGFR and HbA_{1c}. Genome-wide significance ($P<5\times10^{-8}$) is indicated by the red horizontal line. The blue line presents significance $p<10^{-}$.[6] DCS= Diabetes Care System, eGFR= estimated glomerular filtration rate, HbA_{1c}= Hemoglobin A_{1c}, PC= principal component.

Supplementary Table 1. Adjusted models of associations between serum Mg^{2+} concentrations and the lead regional genome-wide significant SNPs.

SNP	chr	Location	Closest gene	% Variance explained	Model 1[3] Beta (mmol/L)	Model 1[3] SE	Model 1[3] P	Model 2[4] Beta (mmol/L)	Model 2[4] SE	Model 2[4] P
rs7894336	10	8026609	*TAF3*	0.53	-0.011	0.002	4.2E-9	-0.011	0.002	1.2E-9
rs11264341	1	155151493	*TRIM46*	0.47	0.010	0.002	1.3E-7	0.010	0.002	1.8E-7
rs10019833	4	77357592	*SHROOM3*	0.37	0.010	0.002	8.9E-7	0.009	0.002	1.6E-6
rs2270860	6	43270151	*SLC22A7*	0.31	-0.010	0.002	1.3E-6	-0.011	0.002	5.6E-8

[1]Allele frequency is for European populations, according to the gnomAD database v3.1.2. (49). [2]Coded alleles are inversely associated with serum Mg^{2+}.

[3]Adjusted for age, sex, eGFR, PC1-3. [4]Adjusted for age, sex, eGFR, HbA_{1c}, PC1-3.

Supplementary Table 2. Loci that display similar effect sizes and an identical direction of the effect on serum Mg^{2+} levels in people with type 2 diabetes (DCS cohort) and in a previous study focused on the general population.

				**Meyer**			**DCS**			**Meta-analysis**					
**SNP**	**EA**	**NEA**	**Gene**	**Beta**[1]	**SE**[1]	**P** [1]	**Beta**	**SE**	**P**	**Beta**	**SE**	**P**	Q_statistic	Q_P	i^2
rs4072037	C	T	MUC1	-0.010	0.001	2.01E-36	-0.009	0.002	6.99E-07	-0.010	0.001	6.87E-29	0.056813	0.811606	0
rs13146355	G	A	SHROOM3	-0.005	0.001	6.27E-13	-0.009	0.002	1.28E-06	-0.006	0.001	2.84E-11	4.102663	0.042816	0.76
rs11144134	T	C	TRPM6	-0.011	0.001	8.21E-15	-0.003	0.004	4.59E-01	-0.011	0.001	2.95E-27	4.452573	0.034849	0.78
rs3925584	C	T	DCDC5	-0.006	0.001	5.20E-16	-0.005	0.002	7.76E-03	-0.006	0.001	5.81E-11	0.137167	0.711114	0
rs10858940	C	T	ATP2B1	-0.007	0.001	1.05E-16	-0.005	0.002	2.00E-02	-0.007	0.001	2.64E-13	0.869574	0.351073	0
rs7197653	C	G	PRMT7	-0.005	0.001	2.02E-06	-0.008	0.003	5.95E-03	-0.005	0.001	1.77E-08	0.788627	0.374516	0
rs2592394	A	G	HOXD9	-0.004	0.001	4.61E-07	-0.002	0.002	2.44E-01	-0.004	0.001	4.05E-05	0.485471	0.485955	0
rs448378	G	A	MDS1	-0.004	0.001	1.25E-08	-0.003	0.002	1.23E-01	-0.004	0.001	2.09E-05	0.252665	0.615205	0
rs4561213	G	T	LUZP2	-0.004	0.001	2.60E-07	-0.003	0.002	1.17E-01	-0.004	0.001	1.97E-05	0.212603	0.644734	0

[1]Data retrieved from Meyer *et al.,* 2010 [1]

chr= chromosome, DCS= diabetes care system, SE= standard error, SNP= single nucleotide polymorphism.

Supplementary Table 3. Loci associated with genetic variability according to GTEx consortium in skeletal muscle tissue.

SNP	chr	Location	Allele frequency[1]	Function	Coded[2]	Closest gene	% Variance explained	Beta (mmol/L)	SE	P	Linked gene[3]	NES[3]	P[3]
rs10795574	10	7948137	0.42	*Intronic*	A>G	*TAF3*	0.45	-0.009	0.002	2.5E-6	*ATP5F1C*	-0.073	9.4E-7
rs760077	1	155178782	0.40	*Missense*	A>T	*MTX1 or THBS3*	0.60	0.010	0.002	4.6 E-06	*THBS3*	-0.211	2.2E-19
rs2870238	4	77373079	0.49	*Intronic*	C>T	*SHROOM3*	0.51	0.010	0.002	4.0E-07	*CCDC158*	-0.200	4.0E-6
rs9394951	6	43350753	0.56	*None*	C>T	None	0.58	0.009	0.002	4.7 E-06	*RPL7L1*	-0.098	4.9E-7

[1]Allele frequency is for European populations, according to the gnomAD database v3.1.2. (49). [2]Coded alleles are inversely associated with serum Mg^{2+}. [3]eQTL of skeletal muscle looked up in the GTEx consortium (50).

Chr= chromosome, GTEx= genotype-tissue expression, Mg^{2+}= magnesium, NES= normalized affect size, PC= principal component, SE= standard error, SNP= single nucleotide polymorphism.

Supplementary Table 4. Loci associated with genetic variability according to human kidney meQTL and eQTM association analyses.

SNP	chr	Location	Coded	Closest gene	Linked gene	CpG	meQTL Beta[1]	meQTL P	eQTM Beta[2]	eQTM P
rs11264341	1	155151493	C>T	*TRIM46*	*EFNA3*	cg23891169	0.578	3.2E-19	-0.136	2.0E-4
rs11264341	1	155151493	C>T	*TRIM46*	*MUC1*	cg13239514	-0.264	8.5E-9	-0.209	3.2E-11
rs10019833	4	77357592	T>C	*SHROOM3*	*STBD1*	cg05861031	-0.687	1.8E-32	-0.196	9.3E-6
rs10019833	4	77357592	T>C	*SHROOM3*	*SHROOM3*	cg12584791	-0.186	2.2E-8	-0.270	1.3E-11
rs2270860	6	43270151	C>T	*SLC22A7*	*POLH*	cg00050375	-0.365	7.3E-12	-0.085	2.6E-5
rs2270860	6	43270151	C>T	*SLC22A7*	*SLC22A7*	cg15360480	0.225	5.5E-7	-0.189	5.9E-5

All data was retrieved from https://susztaklab.com/Kidney_meQTL/index.php (28).

[1]meQTL Beta is reported with respect to the alternate allele.

[2]eQTM Beta is reported as the association between CpG methylation and gene expression.

Chr= chromosome, eQTM= expression quantitative trait methylation, meQTL= DNA methylation quantitative trait locus, PC= principal component, SNP= single nucleotide polymorphism, STD= standard deviation.

Supplementary Table 5. Traits associated with genetic variability according to the Open GWAS database.

SNP	chr	Location	Coded	Population	Trait	Beta	SE	N	P
rs7894336	10	8026609	C>T	European	phosphate	-0.013	0.002	314658	6.3E-8
rs11264341	1	155151493	C>T	European	urea	-0.041	0.002	344052	3.3E-71
rs10019833	4	77357592	T>C	European	cystatin C	0.062	0.002	344264	2.5E-173
rs2270860	6	43270151	C>T	European	cystatin C	-0.032	0.002	344264	1.1E-40

All data was retrieved from the Open GWAS database (29).

Chr= chromosome, SNP= single nucleotide polymorphism.
